# Segmental strain analysis by deformation of a mesh model: comparison of segmental strain metrics and late gadolinium enhancement quantification in myocardial infarction

**DOI:** 10.1186/1532-429X-18-S1-P66

**Published:** 2016-01-27

**Authors:** Alessandro Satriano, Kate Fenwick, Dexter D Waters, Yoko Mikami, Haris Vaid, Bobak Heydari, Derek V Exner, Carmen P Lydell, Andrew G Howarth, James A White, Nowell M Fine

**Affiliations:** 1 grid.410356.50000000419368331Queen's University, Kingston, ON Canada; 2 grid.14709.3b0000000419368649McGill University, Montréal, QC Canada; 3 grid.22072.350000000419367697Division of Cardiology, Department of Medicine, University of Calgary, Calgary, AB Canada; 4 Stephenson Cardiac Imaging Centre, Calgary, AB Canada; 5 Libin Cardiovascular Institute of Alberta, Calgary, AB Canada; 6 grid.22072.350000000419367697Department of Diagnostic Imaging, University of Calgary, Calgary, AB Canada

## Background

The presence and transmurality of posterolateral scar within the left ventricle by late gadolinium enhancement (LGE) imaging has been shown to portend poor response to cardiac resynchronization therapy due to placement of the LV lead in this region. Unfortunately, the use of gadolinium is contraindicated in patients with significant renal dysfunction due to the risk of nephrogenic systemic fibrosis. Alternative measures of regional myocardial non-viability by 4D strain analysis may be highly useful for CRT evaluation in patients with heart failure and renal dysfunction.

## Methods

Ninety-one consecutive patients with known ischemic cardiomyopathy (LVEF 35-50%) underwent cardiac magnetic resonance imaging (CMR) ≥3 months following acute myocardial infarction (MI). Routine, multi-planar long and short axis cine SSFP and matching LGE imaging were obtained for all patients at 1.5 T. GIUSEPPE, an in-house built software, was used to perform 4D strain analysis: the program exploits a 3D mesh obtained from the endocardial and epicardial contours, and computes strain throughout the cardiac cycle by deforming the mesh according to a 4D displacement field reconstructed from the velocity information obtained from feature-tracking. Strains were calculated in Radial (Err), Circumferential (Ecc), Longitudinal (Ell) and Principal directions (Emin and Emax). Strain is available endocardially, epicardially and transmurally. LGE quantification was performed using cvi42 (Circle Cardiovascular, Calgary).

## Results

Mean age for the patient population was 60.6 ± 9.6 years and mean LVEF 42.1 ± 7.1%. Significant correlations were found for all measures of strain versus Global %LGE. Transmural circumferential, longitudinal, radial, minimum principal and maximum principal strain resulted in correlations with %LGE of 0.36, 0.40, -0.31, 0.39 and -0.31, respectively (p < 0.05 for all). Receiving Operator Characteristic analysis showed transmural minimum principal and longitudinal strain to have the best accuracy for detection of segmental LGE>=50% with an area under the curve (AUC) of 0.70 and 0.71, respectively. Sensitivity and specificity at the optimal point were 0.65 and 0.67 for longitudinal strain while they were 0.65 and 0.63 for minimum principal strain. Segmental strain for LGE lower and higher than 50% was significantly different for all strain parameters.

## Conclusions

The present study introduces 4D strain derived from routine, multiplanar cine SSFP imaging as a novel metric for the regional characterization of infarcted tissue in patients with known ischemic cardiomyopathy using percent transmurality by LGE as the gold standard. Future prospective studies using 4D strain analysis by CMR to predict CRT response in patients with renal dysfunction may validate these important findings.

**Figure 1:**
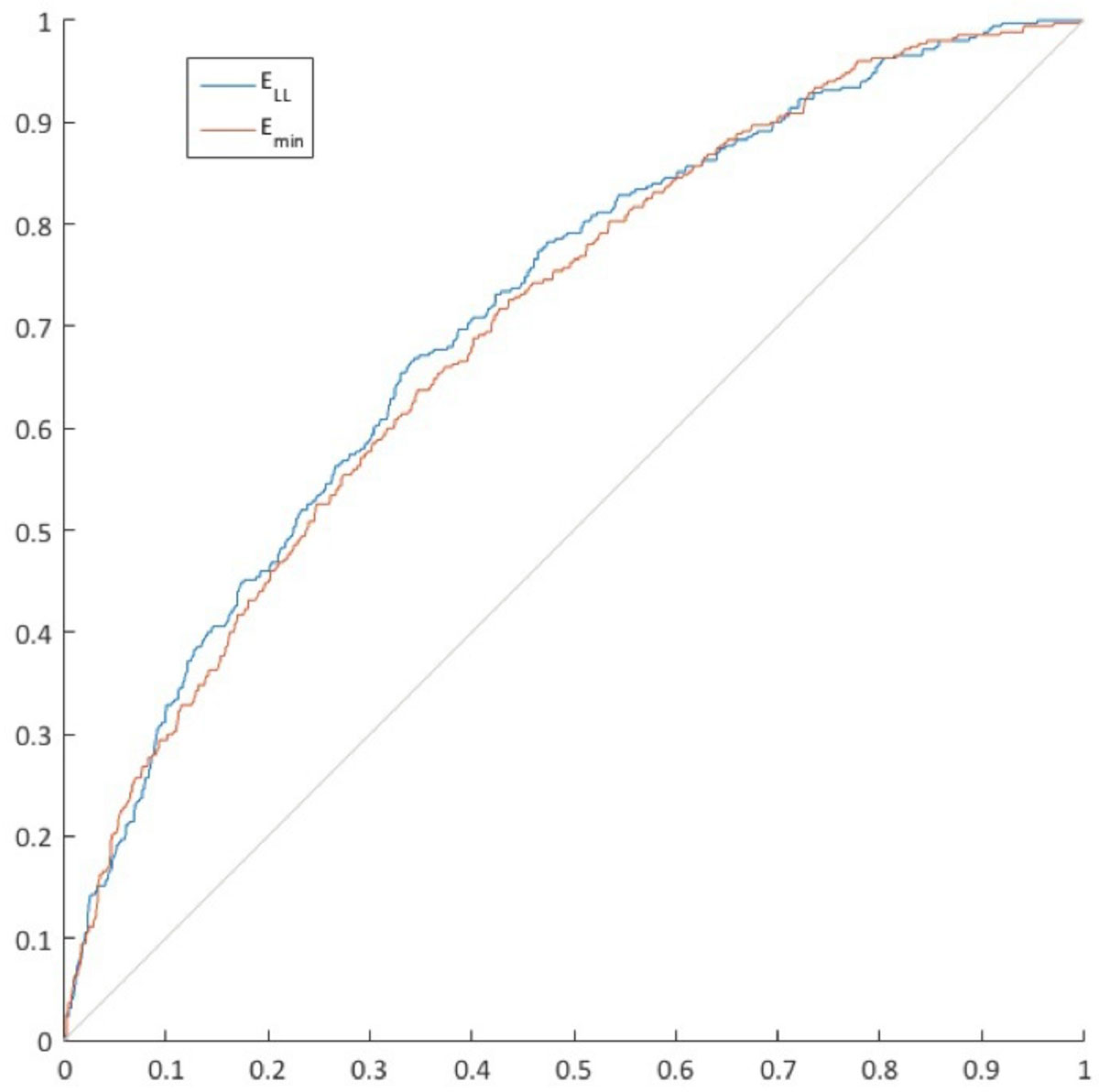
**ROC for the detection of segments with LGE greater than 50% by longitudinal strain (blue) and minimum principal strain (red)**.

